# Species richness both impedes and promotes alien plant invasions in the Brazilian Cerrado

**DOI:** 10.1038/s41598-020-68412-5

**Published:** 2020-07-09

**Authors:** Luciola S. Lannes, Stefanie Karrer, Danielle A. A. Teodoro, Mercedes M. C. Bustamante, Peter J. Edwards, Harry Olde Venterink

**Affiliations:** 1 Department of Biology and Animal Science, São Paulo State University (UNESP), Ilha Solteira, Passeio Monção 226 Zona Norte, Ilha Solteira, SP 15385-000 Brazil; 2 Institute of Integrative Biology, ETH Zürich, Universitätstrasse 16, 8092 Zürich, Switzerland; 3 Department of Ecology, University of Brasilia, Asa Norte, Brasília, DF Brazil; 4 Department of Biology, Vrije Universiteit Brussel (VUB), Pleinlaan 2, 1050 Brussels, Belgium

**Keywords:** Biodiversity, Ecosystem ecology

## Abstract

Worldwide, alien plant invasions have been intensively studied in the past decades, but mechanisms controlling the invasibility of native communities are not fully understood yet. The stochastic niche hypothesis predicts that species-rich plant communities are less prone to alien plant invasions than species-poor communities, which is supported by some but not all field studies, with some very species-rich communities such as the Brazilian Cerrado becoming heavily invaded. However, species-rich communities potentially contain a greater variety of facilitative interactions in resource exploitation than species-poor communities, from which invasive plants might benefit. This alternative hypothetical mechanism might explain why nutrient-poor, species-rich ecosystems are prone to invasion. Here we show that a high species richness both impedes and promotes invasive plants in the Brazilian Cerrado, using structural equation modelling and data from 38 field sites. We found support for the stochastic niche hypothesis through an observed direct negative influence of species richness on abundance of alien invasive species, but an indirect positive effect of species richness on invasive alien plants through soil phosphatase activity that enhances P availability was also found. These field observations were supported with results from a mesocosm experiment. Root phosphatase activity of plants increased with species richness in the mesocosms, which was associated with greater community P and N uptake. The most prominent alien grass species of the region, *Melinis minutiflora*, benefited most from the higher N and P availability in the species mixtures. Hence, this study provides a novel explanation of why species-richness may sometimes promote rather than impede invasion, and highlights the need to perform facilitation experiments in multi-species communities.

## Introduction

The invasion of natural ecosystems by alien plants is recognized as an important component of global environmental change^1–3^, and potentially poses a major threat to biodiversity^4,5^. Getting insight in the mechanisms of alien plant invasions, therefore, has become a major research priority in ecology. Central research questions related to alien plant invasions are: (1) what makes a species invasive?, (2) what are the effects of alien plant invasions on plant communities and ecosystems?, and (3) what makes a native community or habitat ‘invasible’ or vulnerable for invasion?^6–10^. Comparative studies about native and invasive species have shown that many, but not all, invasive species are for instance relatively fast-growing and have a high investment in reproduction^10,11^. The effects of alien invasions on native communities and ecosystem properties are variable and depend on environmental conditions; particularly under nutrient-rich conditions they can become very abundant, forming a threat to native plants, and altering soil conditions^8,12,13^. Two tenets of the invasibility of native communities and habitats are that species-rich plant communities are less invasible than species-poor communities^14–16^, and that nutrient-poor sites are less prone to invasion than nutrient-rich sites^17,18^. However, sometimes also very species-rich communities on nutrient-poor soils are highly invaded^6,19–22^, but this ‘paradox of invasion’ is only poorly understood, so far.

A high diversity of native species is thought to hinder invasion by alien species because it reduces the niche opportunities for the newcomers^14–16^. Although this idea is supported by many observational studies, there are also well-documented exceptions, as cases of species-rich communities becoming invaded by alien species^8,19,20^. These are usually explained by postulating a positive correlation between factors regulating native diversity and those controlling invasion success^16,20^ or to spatial heterogeneity that enables the invasive species to find an unfilled niche^14^. Low nutrient status is thought to make sites less invasible because invasive species can only compete strongly with native species under nutrient-rich conditions^17^. However, these explanations are inadequate to account for cases of alien plant invasions in ecosystems that are both species-rich and very nutrient-poor, such as the Venezuelan Llanos^21^, the Brazilian Cerrado^22,23^, and some Australian and Southern African savannas^21^. The fact that some of these ecosystems are now heavily invaded suggests that other, as yet unknown processes, are also at work.

Plant species diversity can influence resource acquisition in a plant community in various ways. First, more diverse communities are more likely to contain a species with superior adaptive traits for resource acquisition, which is sometimes known as the selectivity effect^24,25^. Second, diverse communities may achieve a higher resource acquisition and biomass production than communities with only a few plant species through complementary use of resources such as N and P^24–27^. Third, one plant species may facilitate the growth of another through resource transfer among species, or through rhizospheric alterations that are beneficial to neighboring plants^28^. A diverse community has a higher chance of having such facilitative interactions and hence of complementary resource use^25,28^. Effects of plant species richness on nutrient availability may also be associated with soil microbes, for instance through increased phosphatase activity^29^. Phosphatases are enzymes released by plants and microbes that hydrolyse organic- into mineral-P, enhancing soil P availability. Little is known about facilitation processes of this type, which potentially involve several species, and it is unknown whether alien invasive species can benefit from them.

We investigated the factors influencing alien plant invasions in Brazilian Cerrado vegetation (Suppl. Figure 1), and explored the nature of complementary facilitation in a mesocosm experiment. The Cerrado is extremely species-rich^30^, and plant growth is often considered to be P-limited^31^. The soils are mainly poor in inorganic^32,33^, though they can be rich in organic P^33,34^, especially in the form of monoesters from which P can be released by the enzyme phosphomonoesterase (phosphatase, hereafter)^34^. Large areas of the Cerrado have been invaded by alien plants, particularly by grasses of African origin, namely *Melinis minutiflora* and *Urochloa decumbens*, which have been introduced as forage grasses in the Cerrado and now dominate large areas posing a threat to native biodiversity^22,23^. Since extractable pools of inorganic N or P in the soil are poor predictors of invasion by African grasses^35^, we hypothesized that these species are more effective than native plants in exploiting organic-P sources, either because they have a higher root phosphatase activity themselves, or because they profit from the phosphatase activity of native plants or from soil microbes. Moreover, based on previous studies showing a positive relationship between soil microbial phosphatase activity and plant species richness^29^, we predicted there would be a positive correlation between plant invasions and species richness, rather than negative correlation predicted by the stochastic niche hypothesis.

## Results and discussion

**Figure 1:**
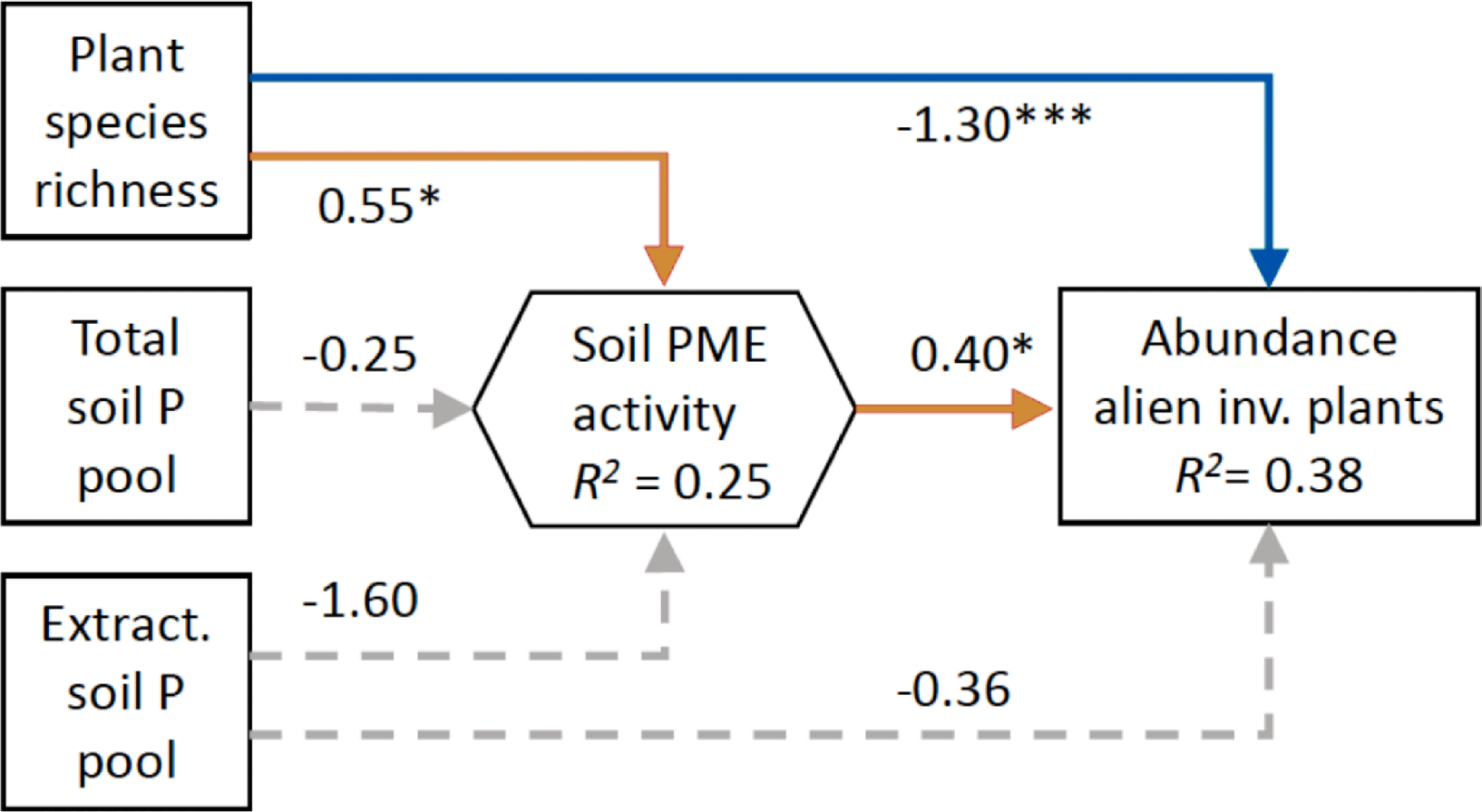
Structural equation model (SEM) showing direct (blue arrow) and indirect (orange arrow) connections between plant species richness and the abundance of alien plants in the Cerrado. The possible connection between species richness and soil phosphatase activity (PME) follows results obtained in the Jena Biodiversity Experiment^29^. Also connections between the total soil P and soil extractable P (Mehlich) pools on soil phosphatase activity, as well as a direct connection between soil extractable P and abundance of alien plants are included in the SEM. Plant variables are recorded on 334-m^2^ plots using the Braun–Blanquet scale, soil parameters are from the top 10-cm soil. Numbers associated with paths between variables are path coefficients presented as standardized values (scaled by the standard deviations of the variables). Solid arrows show significant connections (*p < 0.05, ***p < 0.001), dashed arrows show insignificant connections (*p* > 0.05). Goodness of fit of the SEM: *p* χ^2^ = 0.452 (a good model fit indicating that the fit is clearly not significantly different from the theoretical model).

In a field study conducted at 38 sites in two regions, we measured the abundance of alien invasive species, species richness of the plant community, total and soil extractable pools of P and N, soil phosphatase activity and the root phosphatase activity of nine common plant species. An analysis of the data using structural equation modelling (SEM) revealed no significant relationship between soil extractable-P concentrations and the abundance of alien plants (Fig. 1), despite the fact that previous studies in Cerrado have found P fertilization to promote the invasion of alien species^31^. However, the SEM did find abundance of alien invasive plants to be influenced by native species richness in two contrasting ways. One way was a direct negative relationship between species richness and the abundance of invasive species, which is consistent with the stochastic niche hypothesis and with results of some previous studies^14–16^. This pattern was also observed in a direct regression between the two variables (Suppl. Figure 2). The other way was an indirect and positive effect of species richness that was mediated via phosphatase, suggesting that invasive plants may benefit from organic P released through phosphatase produced by soil microbes and/or plant roots.

**Figure 2:**
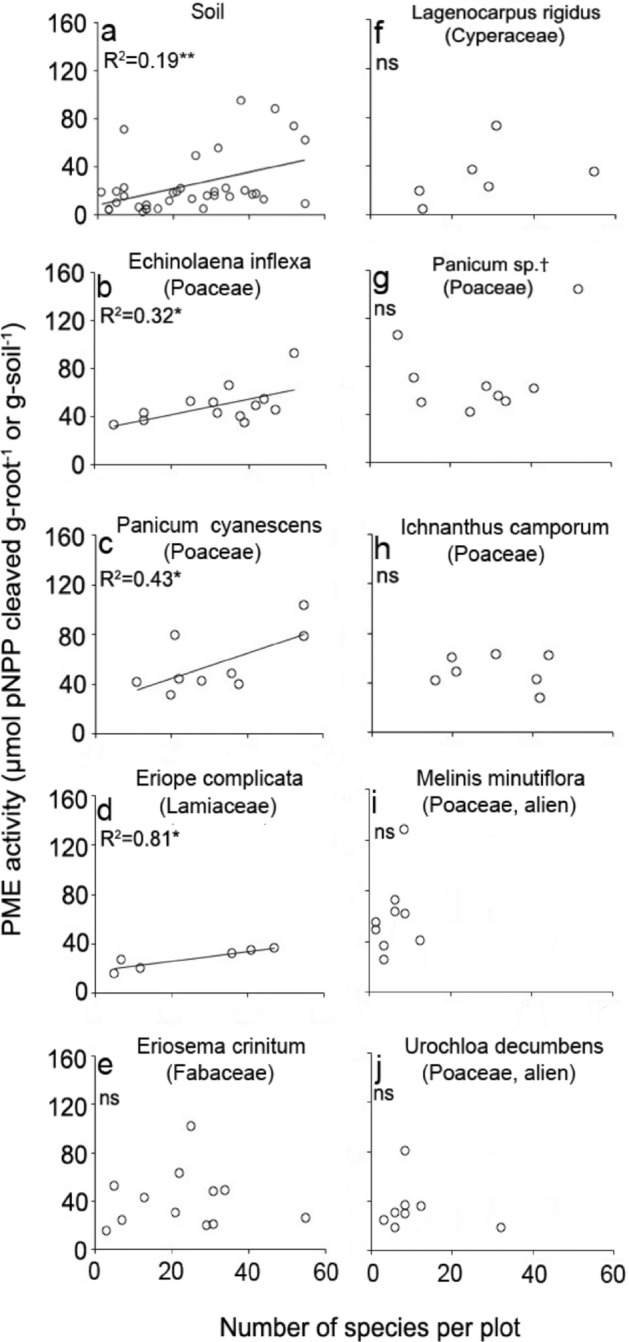
Root phosphatase (PME) activity of common Cerrado plant species, as well as soil PME activity, in relation to species richness of the plant community. (**a**) PME activity in the soil, and (**b**)–(**i**), root PME activity of nine common plant species, in relation to species richness of the vegetation (number of plant species in 4-m^2^). Root and soil samples were collected in 38 sites in the Brazilian Cerrado (five nature reserves in two regions, see Suppl. Figure 1). Only significant (*p* < 0.05) regressions are drawn. †Could not be identified to species level.

A positive relationship between plant species richness and soil phosphatase activity (Figs. 1 and 2a) is consistent with the positive correlation between soil phosphatase activity and manipulated plant species richness observed in the Jena Biodiversity Experiment^29^. The authors attributed the relationship to a positive effect of plant diversity on soil microbial activity and a tight coupling between soil C and P cycling. Because their SEM results did not explain one third of the variation in the data, the authors suggest that there may be a direct link, involving some as yet unknown path, between plant species diversity and soil phosphatase activity^29^. Our finding of a significant positive correlation between root phosphatase activity and species diversity in three of our nine Cerrado species tested (Fig. 2b–j and Suppl. Table 1) not only supports this idea, but suggests a possible mechanism. We conclude, therefore, that under unfertilized conditions alien plants benefit more from the release of organic-P than from a relatively high availability of mineral-P, and that this release is higher in species-rich communities.

To further explore the relationships among plant species richness, plant and soil phosphatase activity, plant P and N uptake, and the abundance of invasive plants, we conducted a mesocosm experiment. We used Cerrado soil in which we planted simple communities composed of one of the two commonest invasive grasses, either *Melinis minutiflora* or *Urochloa decumbens*^22^, combined with the native grasses *Saccharum asperum* and *Setaria poiretiana* and/or the leguminous forb *Stylosanthes guianensis* (3 plants per mesocosm; see Suppl. Figure 3 for the design). To half of the mesocosms, we added a relatively high dose of inorganic P fertilizer (equivalent to 3 g P m^−2^ in the form of dissolved Na_2_HPO_4_). This increased total soil P, but had no effect upon extractable P (Suppl. Figure 4a,b) or on plant P uptake (Suppl. Table 2), indicating that the added phosphate had been immobilized^34^. This rapid immobilization of P in Cerrado soil explains why the addition of inorganic P had almost no effect on traits or on competition of these plants (Suppl. Table 2). It also underlines the importance for Cerrado plants of other P forms, perhaps including mono- or diester-bound organic P and phytic acid. It may be that Cerrado plants have a competitive advantage over soil microbes in accessing these forms of P, whereas microbes are more effective in competing for inorganic phosphate.

**Figure 3:**
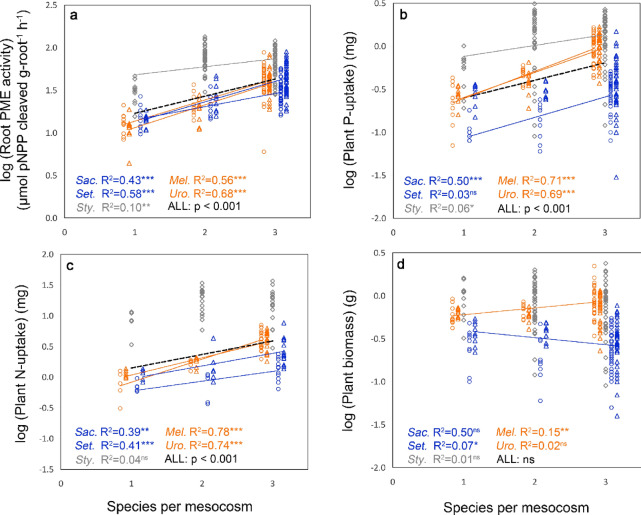
Effects of species richness on root phosphatase (PME) activity, P and N uptake and biomass production of native and alien Cerrado plants in a mesocosm experiment. (**a**) Root phosphatase (PME) activity, (**b**) and (**c**) total P and N uptake from the soil (mg P in 49 days) and (**d**) plant biomass (shoot + root) at harvest (t = 7 weeks) of two alien grasses (*Melinis minutiflora—*orange circles*)* and *Urochloa decumbens—*orange triangles), two native Cerrado grasses (*Saccharum asperum—*blue circles and *Setaria poiretiana—*blue triangles*)* and a native leguminous forb (*Stylosanthes guianensis—*grey losanges) growing in monocultures or in mixtures of two or three species. P fertilization did not have a significant effect on these variables (Suppl. Table 2), therefore the two P treatments were pooled in the regressions. Orange, blue and grey regression lines show significant regressions per species. The dashed black line (ALL) shows the overall effect of the number of species on root PME activity, P and N uptake performed with species identity as random factor (nlme). The design of the experiment is shown in Suppl. Figure 1. Additional statistics are in Suppl. Table 2. To improve visibility of the results in the graphs we subtracted 0.1 or 0.2 from ‘species per mesocosm’ for the alien grasses and added 0.1 and 0.2 for the native grasses.

The results show that species richness had a significant positive effect upon the root phosphatase activity of all plant species, upon P uptake of four out of five species (Fig. 3a,b, Suppl. Table 2), and upon community P-uptake (Suppl. Figure 6g). These were large effects, with the average phosphatase activity and P uptake in the three-species-mixtures being 2.5 times higher than in monocultures (‘All’ in Fig. 3a,b). Unlike the field study, however, there was no correlation between plant species richness and soil phosphatase activity (Suppl. Figure 4e). Hence, for our mesocosm study it is clear that the species richness effect on phosphatase activity was not due to the enzyme activity of soil microbes but to that of the plants themselves, whereas the plant richness effect on soil phosphatase activity in field study could be due to both microbial and plant root activity. Indeed, for at least three plant species we observed a plant species richness effect on root phosphatase under field conditions (Fig. 2b–d). There was also no relationship between species richness and soil extractable-P (Suppl. Figure 4d), supporting the conclusion that the enhanced P uptake in the species mixtures was derived from non-labile P forms such as organic compounds. For two or three of the species studied the differences in root phosphatase activity between treatments were associated with differences in root morphology (specific root branching and surface area) as measured 13 weeks later (Suppl. Figure 5). This association could be causal, since phosphatase activity is known to be highest in the surface cells and apical meristems of roots^36^, but it may also reflect a morphological next to the physiological phosphatase activity response to a greater P demand in the species mixtures.

**Figure 4:**
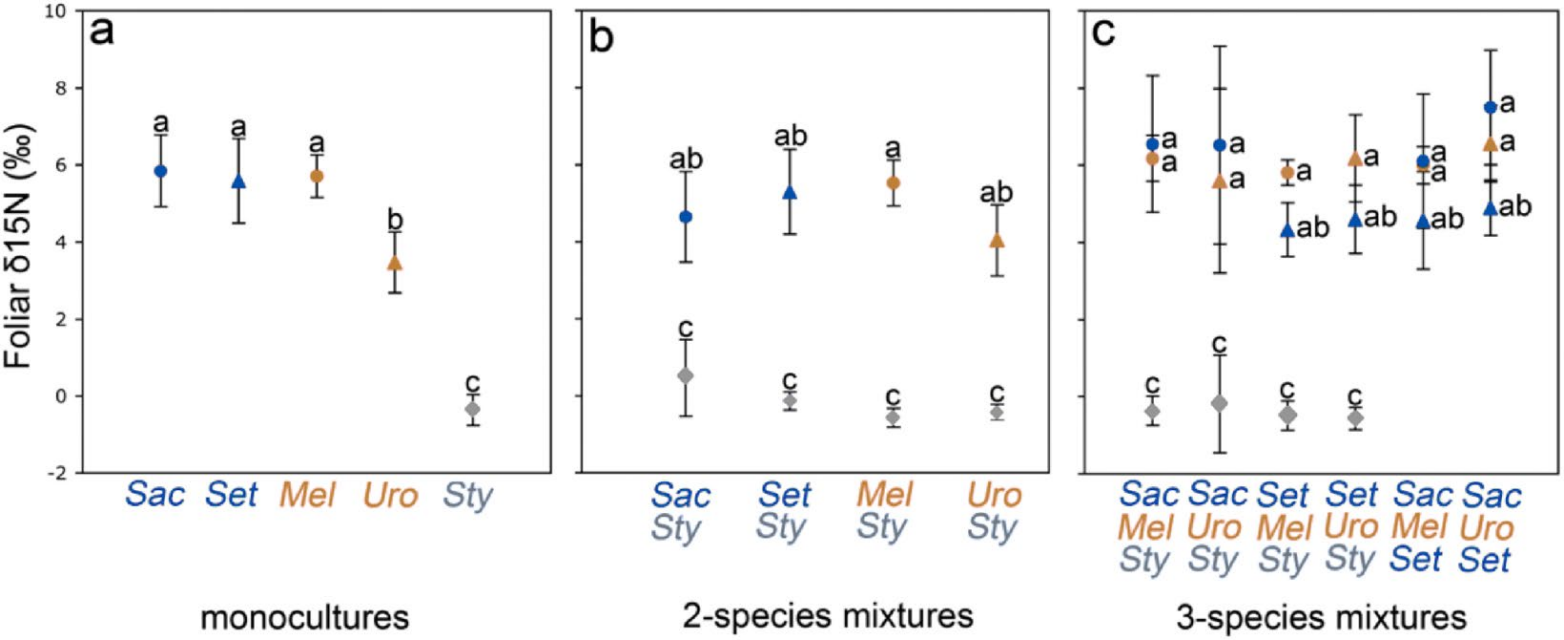
Foliar δ^15^N of native and alien Cerrado plants in a mesocosm experiment. Foliar samples were collected at harvest (t = 7 weeks) of two alien grasses (*Melinis minutiflora—*orange circles*)* and *Urochloa decumbens—*orange triangles), two native Cerrado grasses (*Saccharum asperum—*blue circles and *Setaria poiretiana—*blue triangles*)* and a native leguminous forb (*Stylosanthes guianensis—*grey losanges) growing in monocultures or in mixtures of two or three species. Symbols show mean values (+ SD) of 9–10 replicates for all species in panels a and b, and for *M. minutiflora*, *U. decumbens* and *S. guianensis* in panel (**c**). For panel (**c**) *S. asperum* and *S. poiretiana* had 2–8 and 6–9 replicates, respectively. Only samples from the unfertilized mesocosms were analyzed on foliar δ^15^N. Symbols placed in the same vertical line in panels (**b**) and (**c**) show values from a species combination treatment. Different letters indicate significant differences at the *p* < 0.05 level (Tukey contrasts after Anova Type II).

We also found interspecific differences in foliar δ^15^N in the mesocosm experiment (Fig. 4a). The legume *Stylosanthes guianensis* had the lowest foliar δ^15^N in both monocultures and mixtures, presumably because it obtained most of its N through N_2_ fixation. The alien grass *Urochloa decumbens* had lower foliar δ^15^N than the other three grass species in the monocultures (Fig. 4a), which could be due to the uptake of a different N form^37^, although this difference was no longer detectable in the species mixtures (Fig. 4b,c). Plant N uptake of all four grass species and of the plant communities increased on average with increasing species richness (Fig. 3c, Suppl. Figure 6d). None of the grasses had a significantly lower foliar δ^15^N when growing with *Stylosanthes guianensis* compared to the monocultures (Fig. 4a,b), suggesting that this enhanced N uptake was not due to transfer from the N-fixing species. Instead, the species richness effect can be explained, at least in this short-term experiment, through the complementary use of different N sources (in particular, soil-N vs. atmospheric-N)^17,24,26^. The enhanced N uptake in more diverse communities may also have created a greater demand of other potentially growth-limiting resources, such as P. Hence, species complementarity for different N sources, and enhanced plant N uptake, may have been a driving force for the observed richness effect on plant phosphatase activity (Suppl. Figure 6f,h,i). Similarly, enhanced N uptake in more diverse plant communities has been demonstrated for a broad range of European grasslands^38^. Whether this is the case in the Cerrado plant communities, and whether it is a (co)-driving factor for the observed species richness effect on soil phosphatase activity, and hence on alien plant invasion (Fig. 1), remain to be tested.

Only one species in the mesocosms, the alien grass *Melinis minutiflora,* produced more biomass in mixtures than in monocultures (Fig. 3d). This is the most prominent alien invasive plant in the region of our study^22,31^ (i.e., the region where also the mesocosm soil was collected), and appears to be the species best able to benefit from the additional P and N available in more diverse communities. Its success was not due to it producing a greater root length than other species (data not shown), and presumably reflects greater nutrient use efficiency although we lack the data (on nutrient residence time) to support this. The expansion of this alien species in our mesocosms did not lead to a higher community biomass (Suppl. Figure 6a), but to a shift in species abundances mainly at the cost of one of the native grasses, *Setaria poiretiana* (Fig. 3c). Our results support the conclusion, convincingly demonstrated in a meta-analysis, that native plants often promote the performance of alien plants relative to other co-inhabiting native species^39^. Possibly, the most facilitative native species in our study was the leguminous forb *Stylosanthes guianensis*, for instance because it used a distinct different N source, but species interaction effects on root phosphatase activity, foliar δ^15^N and P or N uptake were also observed in mixtures of only grass species. Facilitation among species may also have been influenced by other soil microbes than N_2_-fixing bacteria, such as mycorrhizal fungi. All species of our study are associated with arbuscular mycorrhiza. We did not record abundances of these fungi, or investigate their role in nutrient transfer among species in our study, but this is worth further investigation.

We note that the variation in species richness in our mesocosm experiment was only in the range of one to three species, whereas species richness in natural plant communities generally go far beyond this number of species. Results of long-term biodiversity experiments such as in Cedar Creek or Jena, as well as model predictions, have shown that already between monocultures and 2, 3 or 4 species mixtures very strong species richness effects are found on ecosystem properties and processes mediated by organism interactions^40,41^. Moreover, the observed patterns in our mesocosm experiment and our field study—where species richness ranged between 1 and 55 per 4-m^2^ species—were very consistent with each other. We therefore presume that the species richness effects on resource facilitation, as we observed in the mesocosm experiment, are very likely to occur in the field as well, and probably even stronger.

## Conclusions

This study has shown that the influence of plant species richness upon alien plant invasions is more complex than previously supposed, especially in very nutrient-poor soils. Species richness can both impede alien plant invasions—in line with the stochastic niche hypothesis^14^—as well as promote the invasion through interspecific facilitation and/or complementarity that enables a severely enhanced exploitation of soil organic-P as well as N. Whether these opposing mechanisms operate simultaneously or at different stages during the invasion process remains an open question. Since many biodiversity hotspots are located on ancient and similarly nutrient-poor soils as the Cerrado^30^, further investigations of this mechanism and its implications for alien plant invasions in other species-rich areas would be worthwhile. This study also has shown that for the understanding of facilitation processes in plant communities, and how these may impact alien plant invasions, multiple species studies are required, whereas most previous facilitation studies are about pairwise interactions^42^.

## Methods

### Field survey

Plant species richness and the abundance of alien plants were recorded in 38 plots located in five nature reserves in the Cerrado (see Suppl. Figure 1), between 15 January and 1 February 2010, which is the peak of the growing season. The Braun–Blanquet scale was used for abundance. In February 2010, three top-10 cm soil cores (5 cm diameter) were randomly collected in each plot and pooled. The soil was analysed on soil extractable P (Mehlich I), ammonium and nitrate in 1 M KCl extracts, water pH, total N and P (Kjeldahl), as well as on soil phosphatase activity following the *p*-nitrophenylphosphate (*p*-NPP) bioassay approach^43^, using 2 g fresh soil in 5 ml *p*-NPP buffered at pH 6. Additionally, roots of the two main native grasses, two main native forbs, and if present all alien species, were excavated and collected at each plot. Root phosphatase activity was measured with the same bioassay approach using 100 mg fresh roots in 5 ml *p*-NPP. Root and soil samples were brought to the laboratory for immediate measurements, and 3–5 analytical replicates were used per plot for the soil and root (*p*-NPP) bioassay which were averaged per plot. Also, at every site aboveground biomass was clipped at 3 cm in three 0.25 m^2^ subplots, and subsequently dried, weighed and analyzed colorimetrically (Quatro AQ2, Seal Analytical, England) on N and P concentrations after Kjeldahl digestion (2040 Digestor Foss Tecator).

Linear regressions between soil or root phosphatase activities and number of species per plot were calculated, as well as Pearson correlations between phosphatase activities and soil extractable N and P, pH and N:P ratio in the vegetation. Structural equation modelling (SEM) was used to determine direct as well as indirect connections between species richness and the abundance of alien plants, following the model described in Fig. 1, with soil phosphatase activity and total-P and extractable-P pools as additional variables. Data were log-transformed when necessary to address model assumptions. Goodness of fit of the SEM (*P* χ^2^) was 0.452, which is a good model fit, and indicates that the fit is clearly not significantly different from the theoretical model. Additional fit variables: Comparative Fit Index (CFI) = 1.000, Tucker–Lewis Index (TLI) = 1.145, Root Mean Square Error of Approximation (RMSEA) = 0.000, Standardized Root Mean Square Residual: 0.022. The relationship between cover of alien plants and species richness was evaluated by means of a generalized linear model for proportional data (glm, binomial family). The dispersion parameter was < 1. Statistical analyses were calculated with R version 3.5.1, except the SEM, which was performed with Stata-IC 15.

### Mesocosm experiment

The experiment was initially designed to investigate the importance of soil and root phosphatase activity for the invasion of alien grasses in the Cerrado, in the presence and absence of a native legume forb. We created monocultures and mixtures of 2 or 3 common Cerrado species grown in mesocosms with Cerrado soil (See Suppl. Figure 3 for an overview of all species combinations). The soil was collected in an uninvaded grassland patch (“campo sujo”) located in a nature reserve (IBGE, see Suppl. Figure 1) in January 2010. All plants were germinated from seeds 6 weeks prior to the experiment. The experiment was carried out in a greenhouse in Brasilia with ten replicates per treatment, starting on 27 January 2010. Half of the mesocosms received a total of 36 mg P-Na_2_HPO_4_, divided in weekly applications.

Plants and soil of five replicated mesocosms were harvested after 7 weeks (15–20 March 2010) for the first set of measurements. Soil and root phosphatase activities of each plant in each mesocosm were measured immediately after harvesting, using the (*p*-NPP) bioassay approach^43^ with 2–3 analytical replicates per root sample which were averaged. Mehlich I extractable soil-P was measured in fresh soil, and total soil P as well as total N and P in the roots and in the aboveground plant tissues after drying at 60 °C and Kjeldahl digestion. Dried plant samples were weighed to determine total biomass per species. Leaves of all plant species in all unfertilized treatments were analyzed on δ^15^N using an elemental analyser (NCS-2500, Carlo Erba) coupled in continuous flow to an isotope ratio mass spectrometer (Optima, Micro-mass).

Plants of another three replicated mesocosms were harvested after 20 weeks (16–18 June 2010). We determined root morphological variables (surface area, number of forks, length, diameter and volume) of each plant using a scanner-based, digital image analysis system (WinRHIZO, Regent Instruments, Canada). Root samples were then dried (60 °C) and root biomass determined. The specific investments in root surface area and production of root tips were calculated by dividing root surface area and number of forks per root dry weight. Half of the mesocosms used for this second part of the project (root morphology) received a total of 51 mg P-Na_2_HPO_4_ during the 20 weeks time.

The effects of number of species per mesocosm (SPECNUM) and P addition on plant biomass, P uptake, N uptake, root phosphatase activity and root morphological traits were analysed per species by means of Anova Type II with SPECNUM as a continuous variable in the model (lm). Additionally, the overall effect of SPECNUM and P addition on these plant traits were analysed by means of a similar Ancova, but with species identity as a random factor (nlme). The effects of SPECNUM and P addition on community biomass, community P and N uptake, and weighted averaged community root phosphatase activity was analysed by means of Anova with SPECNUM as continuous variable (lm). For these community analyses mean values per treatment were used. The weighted average was calculated by taking into account the root biomasses per species as proportions of the community root biomass. The effects of P addition on soil extractable P, total soil P and soil phosphatase activity were measured by means of one-way Anova. Data were log or square-root transformed when necessary to address model assumptions. Statistical analyses were calculated with R version 3.5.1.

## Supplementary information

Supplementary file1
