# Supplementary material for: Species richness both impedes and promotes alien plant invasions in the Brazilian Cerrado
Source: Sci Rep. 2020 Jul 9;10:11365. doi: 10.1038/s41598-020-68412-5 (PMC7347851; doi:10.1038/s41598-020-68412-5)
Supplement: Supplementary file 1 — Supplementary file1 [file 41598_2020_68412_MOESM1_ESM.docx]

**Suppl. Fig. 1– Locations of the field sampling sites in the Cerrado.** Schematic map and geographic coordinates of the 38 field sites sampled in five nature reserves (IBGE, Jardim Botanico de Brasilia, Fazenda Agua Limpa, Pandavas and Fazenda Agua Fria) in two areas of distinct phytophysionomies (Brasilia and Alto Paraiso). The Cerrado area is located almost entirely in Brazil and is delimited by the inner polygon in the map. Source: Modified from Lannes (2012)^44^.


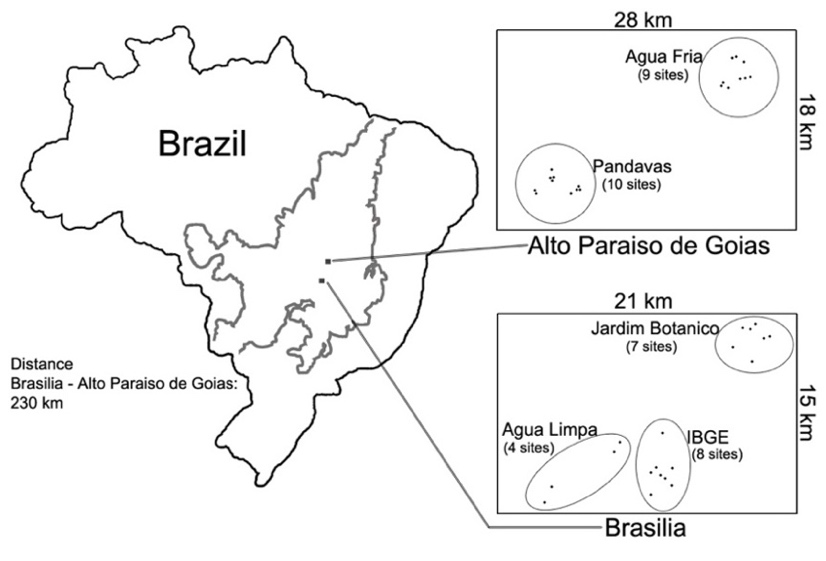


**Suppl. Fig. 2. Cover of alien plants versus species richness in 38 sites of the Brazilian Cerrado** (five nature reserves in two regions, see Suppl. Fig. 1). The regression line is calculated with a generalized linear model (glm) for proportional data (*p*=0.041). A linear regression (lm) was also significant (*p<*0.001).

**
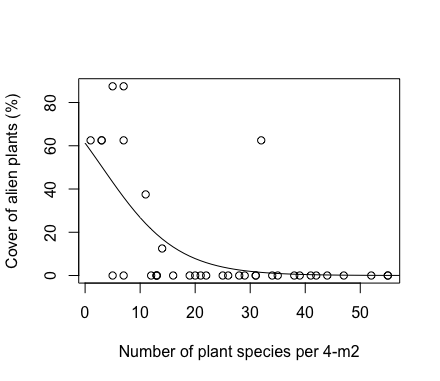
**

**Suppl. Fig. 3 – Experimental design of the mesocosm experiment.** Five perennial plant species (2 alien grasses, 2 native grasses and 1 native legume forb) were grown in various combinations of 1, 2, or 3 species per mesocosm (all with three plants per mesocosm). Mesocosms were filled with 1.7 kg (dry weight) Cerrado soil mixed with washed quartz sand (2:1). For each block, two treatments were applied: control (water-only), and 36 mg P (applied as Na_2_HPO_4_ dissolved in water). All treatments, as shown below, were replicated 10 times for both the control and the P-fertilized treatment. After 7 weeks we harvested 5 replicates for phosphatase activity, P uptake and total biomass. Thirteen weeks after the first harvest, other 3 replicates were harvested for root morphological measurements, with half of them receiving a total of 51 mg P-Na_2_HPO_4_ at the end of the 20 weeks.


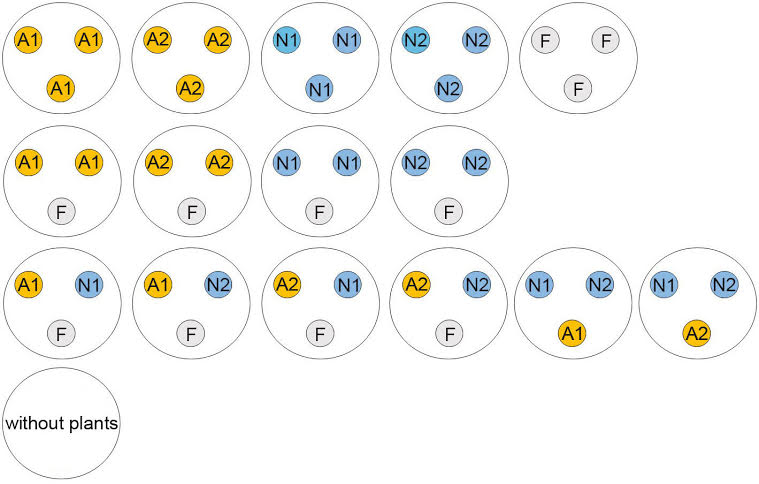


Alien grass species (African species invasive in the Cerrado):

A1: *Melinis minutiflora*

A2: *Urochloa decumbens*

Native Cerrado grasses:

N1: *Saccharum asperum*

N2: *Setaria poiretiana*

Native Cerrado forb (leguminous species):

F: *Stylosanthes guianensis*

**Suppl. Fig. 4 – Effects of mineral P fertilization (a-c) and the number of plant species (d, e) on Mehlich extractable P, total soil P (Kjeldahl) and soil phosphatase (PME) activity in the mesocosm experiment with Cerrado soil.** One-species mesocosms are monocultures of *Melinis minutiflora* (alien grass)*, Urochloa decumbens* (alien grass), *Saccharum asperum* (native grass)*, Setaria poiretiana* (native grass) or *Stylosanthes guianensis* (native leguminous forb)*.* Two and three-species mixtures are made of the possible combinations of these species growing, but two-species of only grasses were not included. The experimental design is shown in Suppl. Fig. 3). The P fertilization was applied as Na_2_HPO_4_ in a dose of 36 mg P per mesocosm. Bars in panels a, b and c show mean values (+ st. error) of 10 replicates, including only the mesocosms without plants. Circles and triangles in d and e show mean values per treatment (species combination and P-treatment) of the unfertilized and P-fertilized mesocosms with plants, respectively, at the time of harvest. Patterns in a, d and e were not significant (*p*>=0.05), *p*-values in a, b and c are from one-way anova (a: df=1, F=1.6; b: df=1, F=4.6; c: df=1, F=0.13). Soil total-P pools were only measured in unvegetated mesocosms.


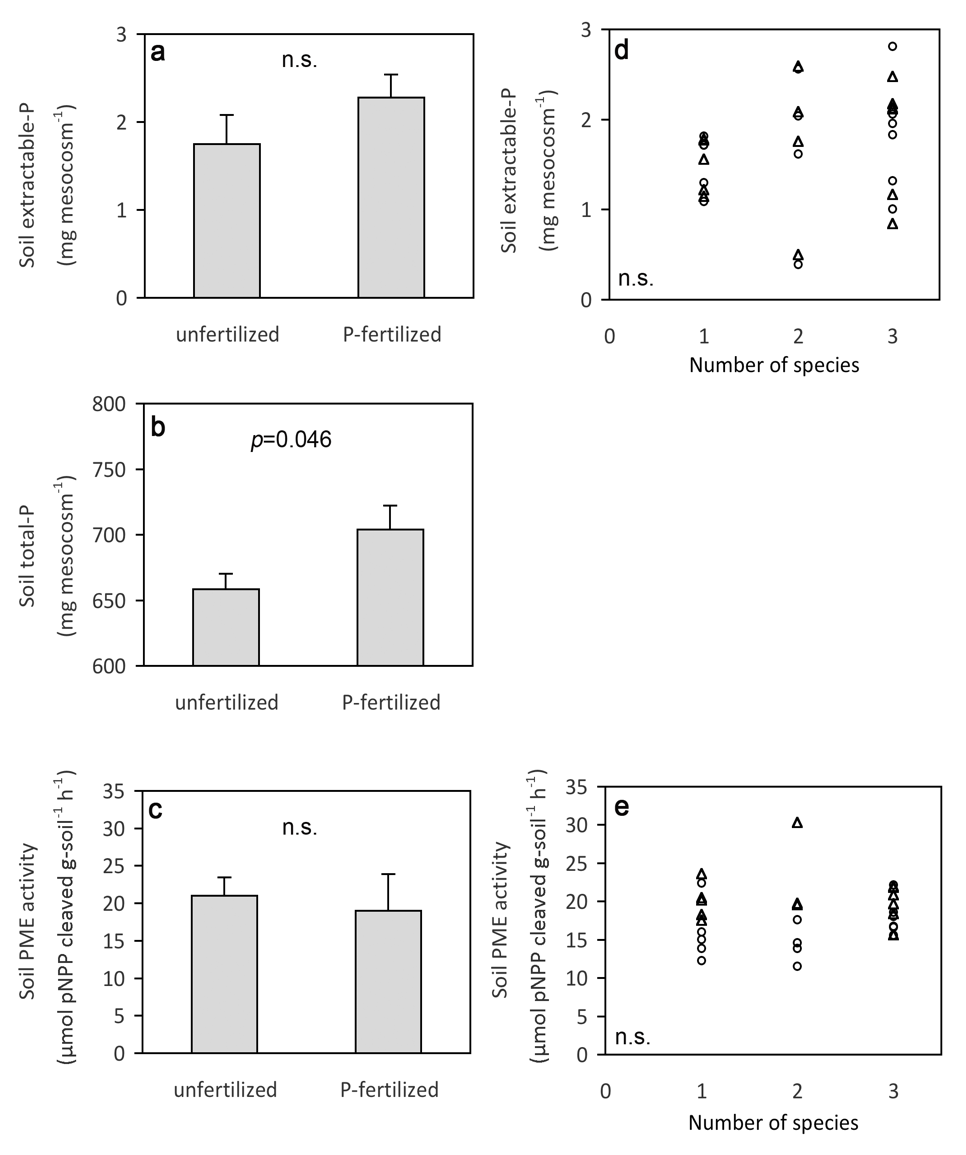


**Suppl. Fig. 5 – Effects of species richness on root morphology of native and alien Cerrado plants in a mesocosm experiment. a,** Specific root surface area (SRSA, root surface area per dry root biomass), **b,** root branching (number of root forks per dry root biomass) at harvest (t=20 weeks) of two alien grasses (*Melinis minutiflora -* orange circles*)* and *Urochloa decumbens -* orange triangles), two native Cerrado grasses (*Saccharum asperum –* blue circles and *Setaria poiretiana -* blue triangles*)* and a native leguminous forb (*Stylosanthes guianensis –* grey losanges) growing in monocultures or in mixtures of two or three species. P fertilization did not have a significant effect on these variables, nor did it interact with species number (Suppl. Table 2), therefore the two P treatments were pooled in the regressions. Orange, blue and grey regression lines show significant regressions per species. The dashed black line (ALL) shows the overall effect of the number of species on the morphological traits, performed with species identity as random factor (nlme). The design of the experiment is shown in Suppl. Fig. 3. Additional statistics are in Suppl. Table 2. To improve visibility of the results in the graphs we subtracted 0.1 or 0.2 from ‘species per mesocosm’ for the alien grasses and added 0.1 and 0.2 for the native grasses.


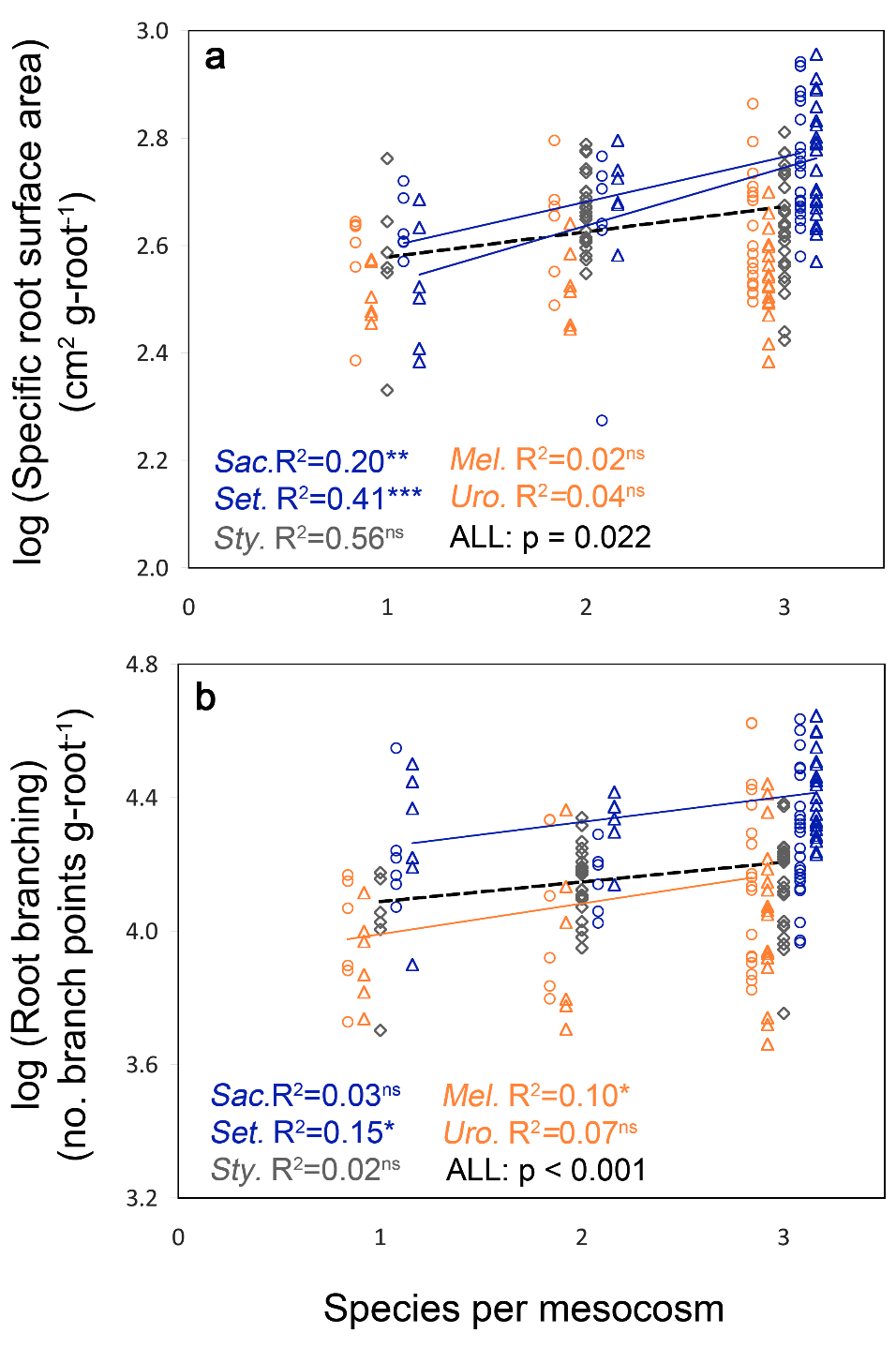


**Suppl. Fig. 6. Community biomass, N uptake, P uptake and weighted average community root phosphatase (PME) activity in relation to species richness of the mesocosms (a, d, g, i), and to each other (b, c, e, f, h, i).** Circles and triangles show mean values per treatment (species combination and P-treatment) of the unfertilized and P-fertilized mesocosms, respectively. The P fertilization did not have significant effects, therefore regressions are shown for the entire data set. n.s. not significant.


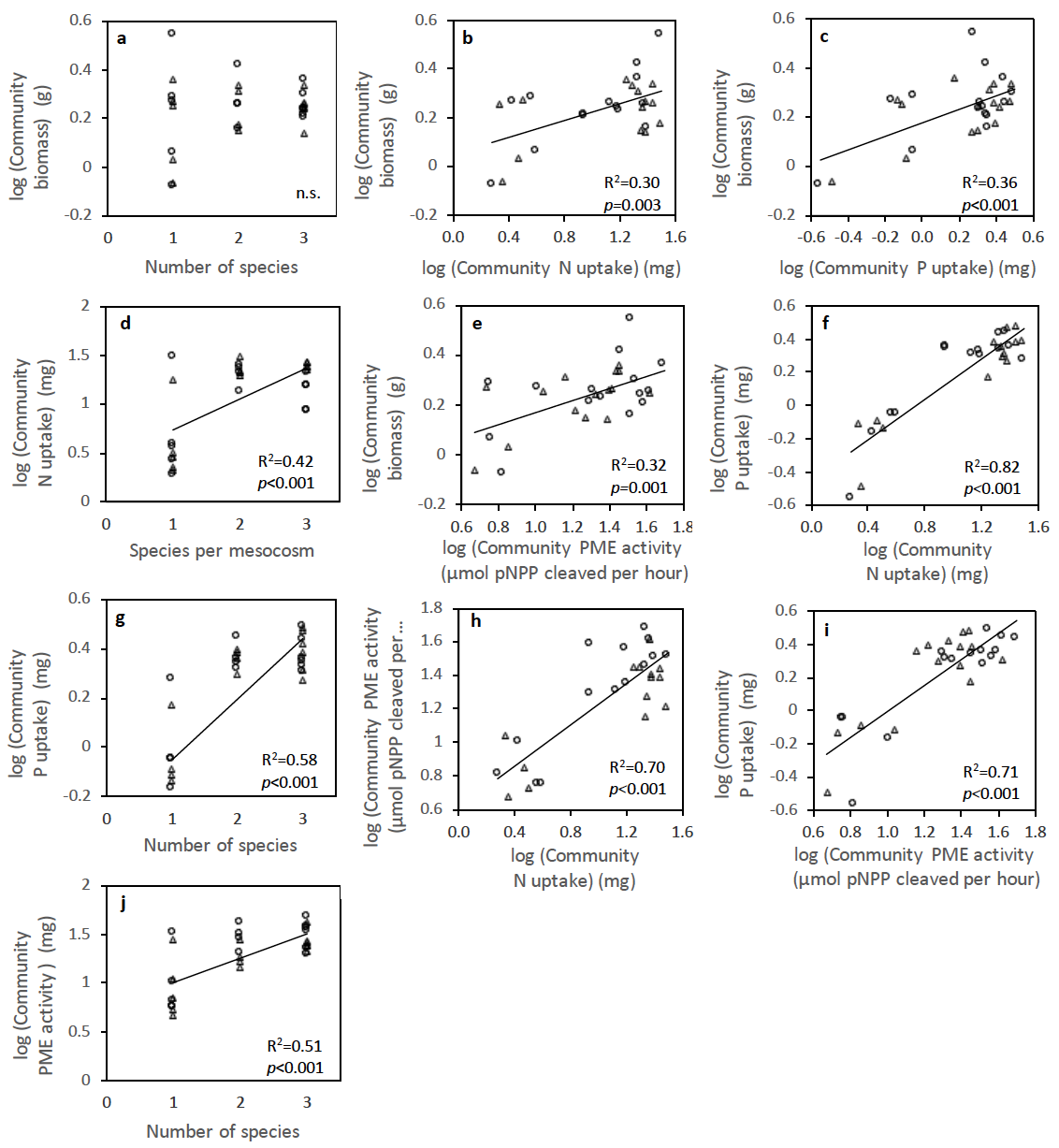
**Suppl. Table 1 – Pearson’s correlation coefficients between soil or root phosphatase activity *versus* some site characteristics.** The number of plants per 4-m^2^ (data of Fig. 2), the N:P ratio in the aboveground vegetation, soil pH, and soil KCl-extractable N (ammonium+nitrate) or Mehlich I extractable P pools. Values in parentheses are number of samples. ^1^ could not be identified to the species level; ^**^ *P*<0.01; ^†^tendency (*P*=0.06).

| **Species** | **Number of species** | **N:P ratio** | **pH** | **Extr. N** | **Extr. P** |
| --- | --- | --- | --- | --- | --- |
| All 9 species (77) | +0.20 | +0.01 | +0.18 | -0.05 | -0.21 |
| *Echinolaena inflexa* (13) | **+0.57**** | -0.29 | +0.07 | +0.03 | -0.27 |
| *Panicum cyanescens* (9) | **+0.66**** | -0.22 | +0.12 | +0.20 | -0.29 |
| *Eriope complicata* (6) | **+0.90**** | +0.44 | +0.51 | -0.32 | -0.60 |
| *Lagenocarpus rigidus* (6) | +0.44 | +0.77 | +0.62 | +0.09 | -0.69 |
| *Panicum sp.^1^* (9) | +0.28 | -0.23 | -0.10 | +0.19 | +0.04 |
| *Ichnanthus camporum* (7) | -0.13 | +0.46 | +0.15 | -0.31 | -0.18 |
| *Eriosema crinitum* (12) | +0.02 | +0.33 | +0.26 | -0.29 | -0.28 |
| *Melinis minutiflora* (9) | +0.26 | +0.12 | -0.01 | -0.03 | -0.45 |
| *Urochloa decumbens* (6) | +0.31 | +0.38 | -0.21 | -0.35 | -0.11 |
| Soil (38) | **+0.44**** | +0.16 | +0.11 | -0.01 | -0.34^†^ |

**Suppl. Table 2. Effects of the number of species per mesocosm, P fertilization and their interaction on biomass production, P-uptake, root phosphatase (PME) activity, root branching and specific root area (SRA) of the five species of the mesocosm experiment (results of Fig. 3 and Suppl. Fig. 5).** F-values and significance levels are shown of Anova (Type II tests). All variables were log-transformed. P treatment was included as factor and species number as a continuous variable in the Anova model. An Anova with species, P fertilizer and number of species could not be calculated because ‘species’ and ‘number of species’ were not independent from each other and this could not be corrected by including mesocosm identity as random factor because the design was not a completely full factorial.

|  | Df | Plant biomass | Plant P-uptake | Plant  N-uptake | Root PME activity | Root branching | Specific root area |
| --- | --- | --- | --- | --- | --- | --- | --- |
| ***Saccharum asperum*** | | | |  |  |  |  |
| No. of species (N)  P fertilization (P)  N * P | 1  1  1 | 0.1 ^ns^  0.1 ^ns^  0.4 ^ns^ | 55.7***  0.1 ^ns^  0.1 ^ns^ | 13.8**  1.5 ^ns^  0.1 ^ns^ | 45.7***  0.9 ^ns^  3.7^†^ | 1.0 ^ns^  0.1 ^ns^  0.9 ^ns^ | 8.4**  2.1 ^ns^  0.1 ^ns^ |
| ***Setaria poiretiana*** | | |  |  |  |  |  |
| No. of species (N)  P fertilization (P)  N * P | 1  1  1 | 4.0*  0.1 ^ns^  0.1 ^ns^ | 0.6 ^ns^  0.3 ^ns^  0.1 ^ns^ | 16.2***  0.1 ^ns^  0.3 ^ns^ | 81.6***  2.0 ^ns^  2.3 ^ns^ | 7.3*  0.7 ^ns^  7.3* | 22.1***  0.1 ^ns^  0.1 ^ns^ |
| ***Melinis minutiflora*** | | | | | | | |
| No. of species (N)  P fertilization (P)  N * P | 1  1  1 | 8.5**  0.2 ^ns^  0.3 ^ns^ | 111.3***  0.4 ^ns^  0.1 ^ns^ | 96.1***  0.9^ns^  0.4 ^ns^ | 61.6***  0.2 ^ns^  2.2 ^ns^ | 3.2^†^  2.3 ^ns^  2.8 ^ns^ | 0.5 ^ns^  0.1 ^ns^  1.0 ^ns^ |
| ***Urochloa decumbens*** | | | | | | | |
| No. of species (N)  P fertilization (P)  N * P | 1  1  1 | 1.0 ^ns^  0.1 ^ns^  0.3 ^ns^ | 104.8***  0.8 ^ns^  0.5 ^ns^ | 70.8***  0.6 ^ns^  0.1 ^ns^ | 98.9***  0.1 ^ns^  1.3 ^ns^ | 1.6 ^ns^  0.1 ^ns^  0.1 ^ns^ | 1.0 ^ns^  0.3 ^ns^  0.1 ^ns^ |
| ***Stylosanthes guianensis*** | | | | | | | |
| No. of species (N)  P fertilization (P)  N * P | 1  1  1 | 0.7 ^ns^  0.9 ^ns^  0.1 ^ns^ | 5.2*  0.1 ^ns^  0.1 ^ns^ | 1.6 ^ns^  0.9 ^ns^  1.7 ^ns^ | 10.0**  0.6 ^ns^  0.1 ^ns^ | 1.1 ^ns^  1.8 ^ns^  0.2 ^ns^ | 0.4 ^ns^  11.9**  0.1 ^ns^ |
| ***ALL (with ‘species’ included as random factor within ‘number of species’)*** | | | | | | | |
| No. of species (N)  P fertilization (P)  N * P | 1  1  1 | 0.1 ^ns^  0.4 ^ns^  0.6 ^ns^ | 17.1***  0.2 ^ns^  0.1 ^ns^ | 18.7***  0.2 ^ns^  0.2 ^ns^ | 36.8***  3.0^†^  0.2 ^ns^ | 11.8***  1.4 ^ns^  0.8 ^ns^ | 5.3*  1.2 ^ns^  0.1 ^ns^ |

*** *P* < 0.001, ** *P* < 01.01, * *P* < 0.05, † *P* < 0.1, ns *P* > 0.1
